# Endoscopic retrieval of a displaced nasocystic catheter from a pancreatic pseudocyst cavity following initial endoscopic ultrasound-guided drainage

**DOI:** 10.1055/a-2807-8775

**Published:** 2026-03-10

**Authors:** Ting Ting Gong, Min Min Zhang

**Affiliations:** 1 Department of Gastroenterology, Ruijin Hospital, School of Medicine, Shanghai Jiao Tong University, Shanghai, China

**Fig. 1:**
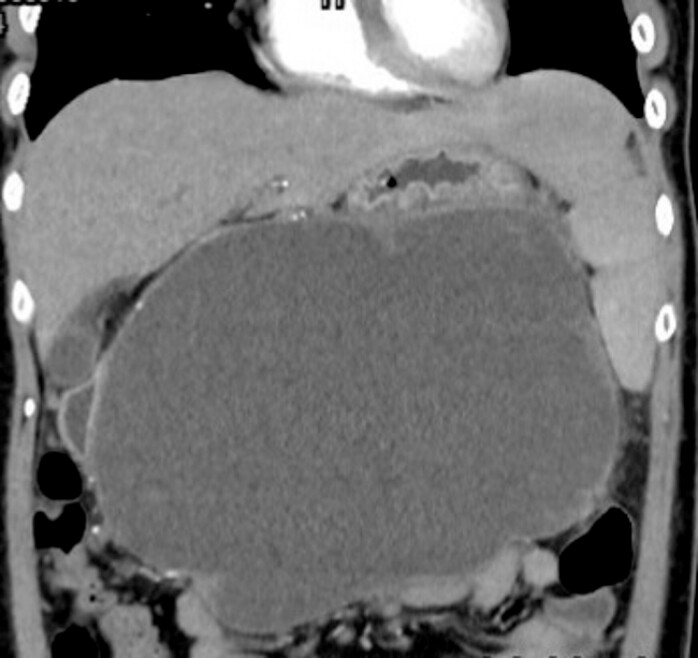
Computed tomography revealed a large pancreatic pseudocyst.

**Fig. 2:**
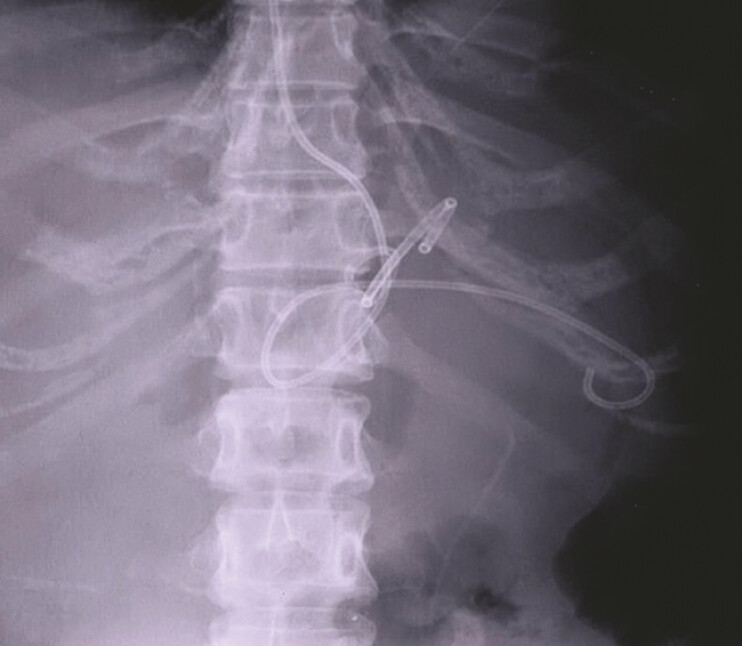
X-ray showed a stent and a nasocystic catheter.

**Fig. 3:**
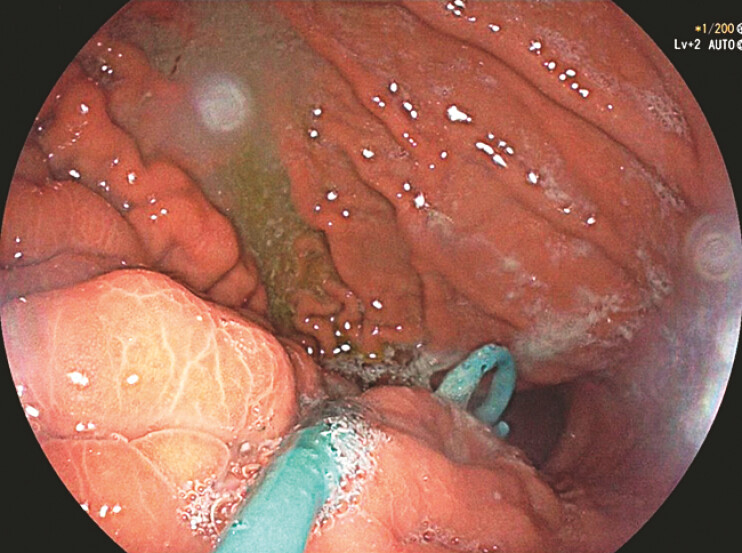
An endoscopic view of a stent and a nasocystic catheter, and the nasocystic catheter was cut in the stomach.

A 36-year-old woman with history of biliary pancreatitis complicated by a large pancreatic pseudocyst ( Fig. 1 ), who presented with abdominal distension, underwent Endoscopic ultrasound (EUS)-guided pseudocyst drainage by the simultaneous placement of a double-pigtail plastic stent and a nasocystic catheter ( Fig. 2 ). The nasocystic catheter inside the stomach was cut under gastroscopy 2 days post-procedure, which served as another plastic stent ( Fig. 3 ).

**Fig. 4:**
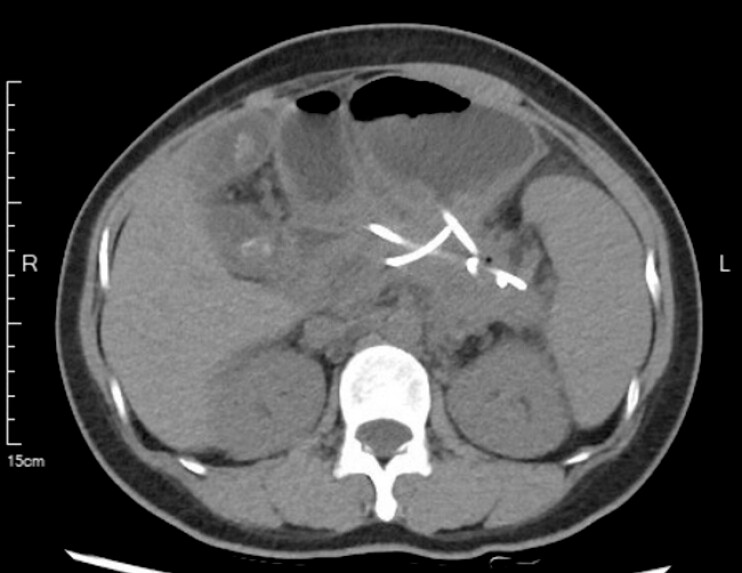
Computed tomography revealed almost disappeared pseudocyst with plastic stents.

**Fig. 5:**
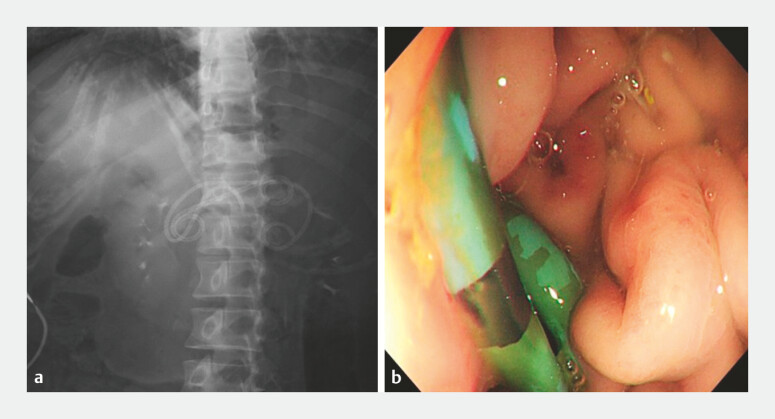
X-ray showed **a** the double-pigtail plastic stent and nasocystic catheter; **b** An endoscopic view of the double-pigtail plastic stent.

Presented 2 months later for the scheduled removal of the two plastic stents ( Fig. 4 ). However, when we inserted the gastroscope into the gastric cavity, we found that the double-pigtail stent was in situ and the nasocystic catheter had migrated completely into the pseudocyst cavity ( Fig. 5 ).

Following failed direct grasping with a snare due to misalignment between the snare forceps and the nasocystic catheter, a duodenoscope was positioned and a guidewire was inserted into the cavity under fluoroscopy. Over this guidewire, an 11 F cholangioscope was advanced into the cavity under combined duodenoscopic and fluoroscopic guidance. The nasocystic catheter was directly visualized within the cavity; however, no compatible retrieval device was available through the cholangioscope channel. The spatial relationship between the catheter and the cholangioscope, correlated with real-time fluoroscopy, allowed precise mapping of the catheter position. A snare forceps was then advanced through the duodenoscope alongside the guidewire, following the same trajectory as the cholangioscope. Under continuous fluoroscopic guidance, the forceps was maneuvered to the predetermined location near the catheter tip. After opening and orienting the jaws, the catheter was successfully grasped and endoscopically withdrawn ( Video 1 ).

Endoscopic retrieval of a displaced nasocystic catheter from a pancreatic pseudocyst cavity following initial endoscopic ultrasound-guided drainage.Video 1

In conclusion, EUS-guided pancreatic pseudocyst drainage has become the standard and safety procedure in many centers for nonsurgical treatment 1 . Stent migration is a known complication 1 2 . This case demonstrates a multimodal endoscopic approach which can successfully solve this problem.

Endoscopy_UCTN_Code_CPL_1AL_2AD
